# Novel Conservative Therapies in Migraine Management: The Impact of Fascia Exercises in a Randomized Controlled Trial

**DOI:** 10.3390/jcm14020539

**Published:** 2025-01-16

**Authors:** Rabia Tuğba Tekin, Hilal Aslan, Veysel Uludağ, Şadiye Gümüşyayla, Gönül Vural

**Affiliations:** 1 Faculty of Physiotherapy and Rehabilitation, Hacettepe University, Ankara 06800, Türkiye; 2 Faculty of Health Science, Physiotherapy and Rehabilitation, Ankara Yıldırım Beyazıt University, Ankara 06800, Türkiye; hilalaslan@aybu.edu.tr; 3 Department of Physiotherapy and Rehabilitation, Faculty of Health Sciences, Duzce University, Duzce 81620, Türkiye; vuludag1365@outlook.com; 4 Department of Neurology, Faculty of Medicine, Ankara Yıldırım Beyazıt University, Ankara 06800, Türkiye; sadiyetemel@yahoo.com (Ş.G.); gonulvrl@gmail.com (G.V.)

**Keywords:** autonomic nervous system, fascia exercises, heart rate variability, migraine, pain management

## Abstract

**Background/Objectives**: Migraine is a complex neurological disorder often associated with autonomic nervous system (ANS) dysfunction. This study aimed to evaluate the effects of fascia exercises on migraine symptoms and explore their potential as a novel conservative treatment approach. **Methods**: A prospective, randomized controlled trial was conducted with 30 migraine patients who were randomly assigned to a treatment group (fascia exercises) or a control group (conventional physiotherapy). Both groups underwent a six-week intervention consisting of two sessions per week. Pain intensity, migraine-related disability, sleep quality, anxiety, depression, heart rate variability (HRV), and patient satisfaction were assessed before and after the intervention using validated scales. **Results**: Significant improvements in pain intensity, attack frequency and duration, migraine-related disability, sleep quality, and anxiety levels were observed in both groups (*p* < 0.05). However, the treatment group demonstrated a more pronounced reduction in depression scores compared to the control group (*p* < 0.05). While no significant changes in HRV parameters were detected in either group, patient satisfaction was significantly higher in the treatment group (*p* < 0.05). **Conclusions**: Fascia exercises represent a promising complementary therapy for migraine management, offering significant improvements in both physical and psychological symptoms. While immediate effects on HRV were not evident, the potential to modulate autonomic balance and address migraine pathophysiology warrants further exploration. These findings highlight the value of fascia exercises as a low-cost, non-invasive approach, emphasizing the need for further research to confirm their long-term clinical benefits and integration into migraine treatment protocols.

## 1. Introduction

Migraine is a common neurological disorder characterized by severe headaches which is often accompanied by symptoms such as nausea, vomiting, and hypersensitivity to light and sound. In some patients, these episodes are preceded by an aura, which manifests as transient neurological disturbances [1,2]. While the exact cause of migraines remains unclear, evidence increasingly points to the dysregulation of the autonomic nervous system (ANS) as a key factor in its pathophysiology. This dysregulation is thought to manifest as an imbalance between the sympathetic and parasympathetic systems, with reduced sympathetic activity observed during interictal periods and heightened sympathetic responses during migraine attacks [3,4,5,6]. This imbalance contributes to vascular dysfunction, heightened pain sensitivity, and impaired homeostasis, collectively exacerbating migraine symptoms.

Several interventions targeting the ANS, such as vagus nerve stimulation and yoga, have shown promise in reducing the frequency and severity of migraine episodes [5,7,8,9]. These therapies work by enhancing parasympathetic activity, mitigating stress responses, and promoting autonomic balance. However, despite their efficacy, these approaches often require specialized equipment or training, which may limit their accessibility. This highlights the need for novel, low-cost, and widely applicable interventions that can modulate the ANS and improve migraine management outcomes.

Fascia, a complex network of connective tissue that envelops and connects muscles, nerves, and blood vessels, has emerged as a promising target for such interventions. Fascia is known to play a significant role in pain modulation, circulation, and ANS regulation [10,11,12]. Recent studies have demonstrated that mechanoreceptor stimulation within the fascia can influence autonomic outflow, reduce sympathetic overactivity, and improve parasympathetic balance [13,14,15]. This is supported by research in mechanotransduction, which suggests that physical manipulation of fascial tissue can activate sensory neurons and modulate pain pathways [13,16]. Techniques such as myofascial release and related exercises have been shown to alleviate musculoskeletal pain and improve functional outcomes, providing further evidence of fascia’s therapeutic potential [14,15,16,17].

Despite these promising findings, the role of fascia-focused interventions in neurological disorders, including migraine, remains underexplored. Existing studies have largely concentrated on musculoskeletal applications, leaving a significant gap in understanding their effects on conditions involving the ANS. Addressing this gap, the present study investigates the impact of fascial exercises on migraine symptoms, particularly their potential to modulate autonomic regulation and reduce pain severity. By leveraging the interplay between fascial dynamics and ANS activity, this research aims to establish a foundation for incorporating fascia-based therapies into migraine management. The study’s exploratory design seeks to provide preliminary insights and stimulate further research into the efficacy and mechanisms of fascia-targeted interventions.

## 2. Materials and Methods

### 2.1. Study Design

This study was designed as a prospective, randomized controlled trial to compare the effects of fascia exercises and conventional physiotherapy in patients with migraine. The study was registered on clinical.gov.tr, and a clinical trial number was obtained (NCT06231615). Participants were randomly assigned to one of two treatment groups using simple random allocation. At the initial interview, participants were asked to select one of two opaque envelopes containing the group names. To ensure allocation concealment, the randomization process was conducted by an independent person not involved in the treatment procedures. Participants were blinded to the interventions they received.

### 2.2. Participants

**Figure 1:**
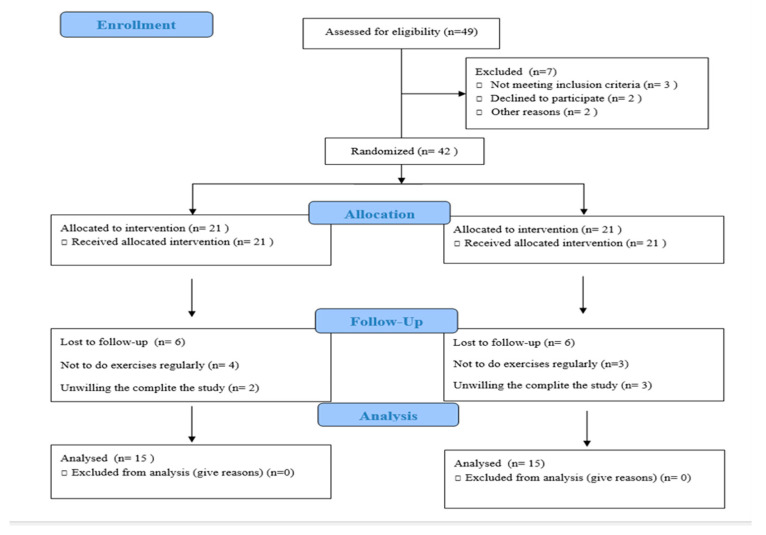
CONSORT flow diagram depicting participant recruitment, randomization, and study completion.

A total of 49 migraine patients who met the inclusion and exclusion criteria were screened, and 30 eligible participants completed the study (Figure 1). Before starting the study, participants were verbally informed about the study’s purpose, content, duration, and the evaluations and treatments to be performed. Ethical approval was obtained from the Ankara Yıldırım Beyazıt University Health Sciences Ethics Committee (approval date: 7 April 2022, protocol no: 06) in compliance with the Declaration of Helsinki. Informed consent was obtained from all participants. Evaluations were conducted at Ankara Bilkent City Hospital, where the treatment program was also implemented. The necessary permissions to execute the treatment program were obtained from the hospital. Participants were included in the study if they had a diagnosis of migraine for at least one year, were aged between 18 and 65 years, experienced at least five migraine attacks per month, had a history of migraine onset before the age of 50, and provided informed written consent to participate. Participants were excluded if they had headaches due to organic or secondary causes such as subarachnoid hemorrhage, hypertension, cerebral embolism, or thrombosis. Additional exclusion criteria included receiving acupuncture treatment within the last six months, having a history of bleeding diathesis or anticoagulant therapy, being pregnant or breastfeeding, and having a history of malignancy, depression, or antidepressant treatment. Further exclusion criteria involved caffeine consumption in the last 4 h, tobacco use within the last 48 h, drug or alcohol use in the last week, or eating within two hours prior to evaluation. Participants were also excluded if they were unable to move independently, had uncontrolled medical conditions, recent surgeries, or chronic cardiovascular diseases, or were using medications or had conditions that affected the autonomic or immune system, such as beta-blockers, steroids, or TNF-alpha inhibitors.

Power analysis was conducted using the G*Power program, which determined that a total of 30 participants, with 15 individuals in each group, would be sufficient to achieve a 5% margin of error (*p* = 0.05) and 80% study power. Effect sizes of 0.5 and variability estimates based on previous studies in migraine interventions were used for the calculation [16]. While this sample size is appropriate for an exploratory study, it represents a limitation for detecting smaller effect sizes. Future studies should aim for larger cohorts and consider different effect sizes and variability estimates to improve statistical robustness and generalizability.

### 2.3. Evaluations

Patients were evaluated by a blinded researcher (R.R) before and after treatment. In addition to demographic data and descriptive information on migraine characteristics, “number of medications” was used for migraine pain, a visual analog scale (VAS) was used for pain levels, the Migraine Disability Scale (MIDAS) was used for migraine-related disability levels, the Pittsburgh Sleep Quality Index (PSQI) was used for sleep quality, the Hospital Anxiety and Depression Scale (HADS) was used for anxiety and depression levels, and a Polar H10 model heart rate monitor was used for HRV. Participants’ satisfaction was also evaluated after the treatment. Participants’ satisfaction was also evaluated after the treatment using a validated 5-point Likert scale.

### 2.4. Pain Intensity

Participants’ pain intensity was measured using the VAS, separately for day- and night-time periods. On a 10 cm straight line, the patients were presented with the starting point as “no pain” and the end point as “unbearably severe pain”, and were asked to mark the level corresponding to the intensity of pain they felt between these two points [17].

### 2.5. Level of Disability Due to Migraine

The Migraine Disability Scale (MIDAS), which is used to evaluate the level of disability due to headache, investigates the effect of headache in the last 3 months and consists of five items. The MIDAS score was obtained by calculating the days that reduced or completely prevented the patients’ work and schoolwork, housework, and the time spent with family and friends [18].

### 2.6. Sleep Quality

The Pittsburgh Sleep Quality Index (PSQI), a self-report scale assessing sleep quality and sleep disturbance over a one-month period, consists of 7 components: subjective sleep quality, sleep latency, sleep duration, habitual sleep efficiency, sleep disturbance, sleep medication use, and daytime dysfunction. Each item is evaluated on a 0–3-point scale and the sum of the 7 component scores constitutes the total PSQI score. The total score has a value between 0 and 21, and a high total score indicates a poor sleep quality [19].

### 2.7. Heart Rate Variability

All measurements were performed in a quiet environment, under thermoneutral conditions (22–24 °C and 40–60% relative humidity), after the subject had rested for 15 min in a seated position. Recordings were taken in the supine position with spontaneous breathing for 5 min to ensure consistency and minimize variability. A Polar H10 model heart rate monitor (H10, Polar Electro, Kempele, Finland) was chosen for its high accuracy and validation in previous HRV studies. The electrodes on the Polar H10 Bluetooth chest strap were moistened with water at room temperature before placement to ensure optimal conductivity and accurate signal detection. The sensor was positioned precisely on the xiphoid process of the sternum and secured using adjustable Velcro straps to prevent movement artifacts during the measurement. The Polar H10 chest strap automatically connected to the Elite HRV© system (Elite HRV, Asheville, NC, USA), and the collected data were analyzed using the Elite HRV smartphone application. This protocol follows manufacturer guidelines and adheres to standardized HRV research methodologies, ensuring reliable and reproducible results [20,21].

### 2.8. Anxiety and Depression

Anxiety and depression levels of the patients were evaluated with the Hospital Anxiety and Depression Scale (HADS). The scale consists of 14 items, 7 of which evaluate anxiety and the other 7 evaluate depression, and these questions are evaluated with a 4-point Likert scale based on a scoring system between 0 and 3 [22].

### 2.9. Patient Satisfaction

Satisfaction with the results of fascia exercises was treated as the primary outcome measure. Overall satisfaction with fascia exercises for migraine was assessed using a 5-point Likert scale. Patients were asked the question “Which of the following options best describes your overall satisfaction with fascia exercises for migraine?” and the scoring was as follows; 1: very satisfied, 2; quite satisfied, 3; neither satisfied nor dissatisfied, 4; somewhat satisfied, 5; not satisfied at all [23].

### 2.10. Treatment Protocol

The treatment program was conducted over six weeks, with two sessions per week lasting 45 min each. Both groups underwent head-neck exercises for migraine pain and relaxation exercises combined with breathing techniques. In the treatment group, fascia-specific exercises targeting appendicular, axial, meningeal, and visceral fascia were additionally applied. All interventions were performed by a blinded therapist.

### 2.11. Conventional Physiotherapy Program

Participants in the conventional physiotherapy group followed a structured exercise protocol designed to improve mobility, reduce muscle tension, and promote relaxation. The program included active range-of-motion exercises for the head and neck in all directions, such as flexion, extension, lateral flexion, and rotation, with 10 repetitions performed for each movement. These exercises were performed at a slow and controlled pace to ensure proper form and minimize discomfort. In addition, self-stretching exercises targeting the upper trapezius, levator scapulae, and sternocleidomastoid muscles were conducted in a seated position. Each stretch was held for 20–30 s and repeated three times. Diaphragmatic breathing exercises were incorporated to activate the parasympathetic nervous system, with participants practicing controlled inhalation and exhalation cycles while focusing on diaphragmatic movement. These breathing exercises were performed for five minutes per session. The protocol also included relaxation techniques involving rhythmic contraction and relaxation of muscles, progressing from distal to proximal muscle groups. Each muscle group was contracted for five seconds and relaxed for 10 s to encourage the release of tension. Lastly, manual massage techniques were applied to the neck and facial areas, focusing on key regions such as the suboccipital muscles, temporalis, and masseter. These massages were performed in both supine and prone positions and lasted approximately 10 min per session. Each session lasted 45 min, with exercises tailored to the participants’ comfort levels to ensure adherence and prevent fatigue.

### 2.12. Fascial Pattern Exercises

**Figure 2:**
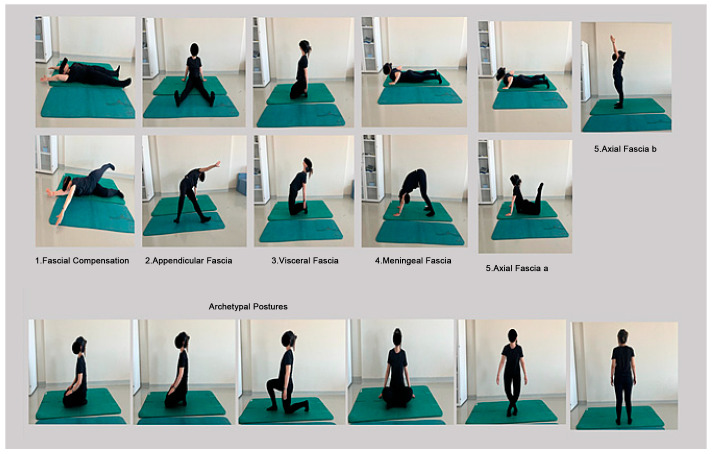
Illustration of fascial pattern exercises and archetypal postures applied during the study.

The fascia-specific exercises were performed on a mat in various positions targeting the appendicular, axial, meningeal, and visceral fascia. Each movement was repeated five times, with a 10 s hold at the end of each movement, followed by a slow return to the starting position. Archetypal postures were practiced at the beginning and end of the sessions and were maintained for 3 to 15 min to enhance fascial mobility. Breaks were allowed if discomfort occurred, and minor modifications were made to accommodate participants’ needs (Figure 2).

### 2.13. Statistical Analysis

SPSS software version 26 was used for the statistical analysis of the data. Categorical variables are presented as number and percentage, and continuous variables are reported as mean ± standard deviation. The Shapiro-Wilk test was used to determine the normal distribution of numerical variables. The Wilcoxon test was used to determine the changes within a group and the Mann–Whitney U test was used to determine the differences between two groups. The chi-square test was used to determine the differences between the two groups to compare the proportions. Effect size (ES) measurement was used to determine the magnitude of change in the evaluated parameters. An ES value between 0.20 and 0.50 was considered ‘weak’, an ES value between 0.51 and 0.80 was considered ‘moderate’, and an ES value greater than 0.80 was considered ‘strong’. *p* < 0.05 was considered statistically significant, and all results are expressed with 95% confidence intervals.

## 3. Results

The mean age of the participants was 31.83 ± 11.92 years, and all participants were female. There were no significant differences in age, height, weight, and BMI between the control and treatment groups (*p* > 0.05). Among the participants, 14 (46.7%) had migraine with aura, and 21 (70%) reported no family history of migraine. Migraine characteristics were also similar between the groups, with no significant differences observed (*p* > 0.05). These data are detailed in Table 1 and Table 2.

Baseline comparisons showed no significant differences between the control and treatment groups regarding the number of medications used for migraine pain, weekly attack frequency, attack duration, or the intensity of daytime and night-time pain (*p* > 0.05). After treatment, the treatment group exhibited significant reductions in the number and duration of attacks, as well as in VAS Day and VAS Night scores (*p* < 0.05). The control group also demonstrated significant decreases in the number of medications used, attack frequency, attack duration, and both VAS Day and VAS Night scores (*p* < 0.05). However, no significant differences were found between the groups when comparing changes in these variables before and after treatment (*p* > 0.05). These findings are summarized in Table 3.

Disability levels, sleep quality, anxiety, and depression scores were comparable between the groups at baseline (*p* > 0.05). After treatment, significant improvements were observed in both groups for the MIDAS, PSQI, and the anxiety component of the HADS (*p* < 0.05). Additionally, the treatment group showed a significant reduction in depression scores compared to the control group (*p* < 0.05), highlighting the potential psychological benefits of fascia exercises. No significant changes were found in the 24-hr-MQoLQ scores in either group, and nor were there significant differences between groups in the changes in these variables before and after treatment (*p* > 0.05). These results are presented in Table 4.

Baseline HRV parameters were similar between the groups (*p* > 0.05). Following treatment, a statistically significant improvement was observed in mean RR values within the control group (*p* < 0.05). However, no significant changes were detected in other HRV parameters within or between groups (*p* > 0.05), suggesting that the intervention may not have an immediate effect on autonomic regulation. The differences in HRV parameters are summarized in Table 5.

After the intervention, patient satisfaction was significantly higher in the treatment group that received fascia exercises compared to the control group (*p* < 0.05). This indicates a higher perceived benefit and acceptability of fascia exercises as an intervention, despite the lack of significant group differences in some objective measures. These results are presented in Table 6.

**Figure 3:**
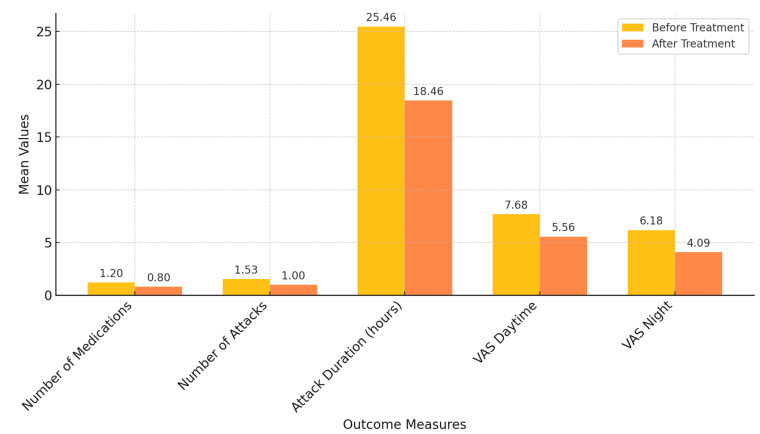
Comparison of outcome measures before and after treatment in the treatment group.

This bar chart illustrates the mean values of key outcome measures, including the number of medications used per day, the number of migraine attacks, attack duration (in hours), and visual analog scale (VAS) scores for daytime and night-time pain, both before and after the intervention in the treatment group. Significant improvements were observed across all measures after the fascia exercise intervention, demonstrating its potential efficacy in reducing migraine-related symptoms. Comparison of the outcome measurements before and after treatment in the treatment group is given in Figure 3.

## 4. Discussion

This study is one of the first randomized controlled trials to investigate the effects of fascia exercises on migraine symptoms, addressing a notable gap in the literature. The findings suggest that fascia exercises can positively influence pain intensity, migraine-related disability, sleep quality, and psychological symptoms. However, these results should be interpreted with caution due to the exploratory nature of the study and its methodological limitations.

The reductions in pain intensity, attack frequency, and duration observed in the treatment group are consistent with the physiological mechanisms associated with fascia manipulation. Fascia, as a highly innervated connective tissue, plays a key role in modulating autonomic nervous system (ANS) activity and pain perception. Mechanoreceptor stimulation during fascia exercises may reduce sympathetic overactivity, promote parasympathetic modulation, and enhance local vasodilation, which collectively contribute to pain relief. These findings align with previous studies demonstrating the efficacy of myofascial release and related techniques in pain management [24,25]. Similar benefits have been observed in interventions such as yoga, which also target ANS regulation and have been shown to reduce migraine severity [26,27]. While these mechanisms are plausible, further research is needed to validate these pathways and their relevance to migraine pathophysiology.

Heart rate variability (HRV), an important marker of ANS function, did not exhibit significant changes during the six-week intervention period. This finding suggests that the intervention duration may not have been sufficient to elicit measurable autonomic adaptations. Previous studies have demonstrated that longer intervention durations or higher session frequencies are often necessary to enhance parasympathetic tone and reduce sympathetic dominance [9]. The absence of significant HRV changes underscores the need for extended follow-up periods in future research to better capture the autonomic effects of fascia exercises. Additionally, exploring complementary biomarkers, such as vagal tone or inflammatory cytokines, could provide a more comprehensive understanding of how fascia exercises influence the ANS and migraine symptoms [28,29,30,31].

Improvements in psychological outcomes, particularly depression, were more pronounced in the treatment group compared to the control group. Depression is a common comorbidity in migraine, exacerbating the condition’s impact on quality of life and functional capacity [32]. The reduction in depression scores observed in this study suggests that fascia exercises may provide psychological benefits in addition to physical symptom relief. This dual impact highlights the potential of fascia exercises as an integrative approach that addresses both the physical and emotional burdens of migraine. These findings are consistent with prior studies demonstrating that structured physical activity can alleviate psychological distress and improve overall well-being in patients with chronic conditions [33,34].

The results also indicate significantly higher patient satisfaction levels in the fascia exercise group. This suggests that the structured nature of the intervention may have provided a sense of control and predictability, which are critical for managing chronic conditions like migraine. According to the biopsychosocial model, chronic pain is influenced by cognitive, emotional, and behavioral factors, and interventions that actively engage patients can reframe their pain experiences positively [35,36]. The combination of physical activity and targeted myofascial manipulation likely contributed to the favorable perception of the intervention.

Despite its strengths, this study has several limitations. The relatively small sample size limits the statistical power and generalizability of the findings. Additionally, the six-week intervention duration precludes an evaluation of long-term outcomes and limits the ability to detect significant changes in autonomic markers, such as HRV. Another methodological limitation is the randomization process, which was conducted using sealed opaque envelopes rather than a computer-generated randomization method. While this approach was practical and cost-effective, it may have introduced allocation bias. Future studies should implement computer-based randomization methods to enhance allocation concealment and methodological rigor. The absence of a placebo-controlled group also introduces the possibility of non-specific effects influencing the results. Finally, the reliance on quantitative measures without incorporating qualitative feedback limits the depth of interpretation. Future research should address these limitations by including larger cohorts, longer intervention periods, placebo-controlled designs, and qualitative methodologies to provide more robust evidence and insights.

## 5. Conclusions

This study provides preliminary evidence supporting the use of fascia exercises as a potential intervention for managing migraine symptoms. The findings suggest that these exercises may alleviate physical symptoms such as pain intensity and migraine-related disability while also addressing psychological aspects, including anxiety and depression. However, the results must be interpreted cautiously due to the exploratory nature of the study and its methodological limitations.

Fascia exercises represent a promising, non-invasive approach that combines physical activity with targeted connective tissue manipulation. While this study observed notable improvements in pain and psychological symptoms, no significant changes were detected in heart rate variability (HRV), a marker of autonomic nervous system function. This highlights the need for further research to understand the mechanisms underlying these effects, particularly their potential impact on autonomic regulation.

Future research should aim to validate these findings through larger-scale, randomized controlled trials with extended intervention durations and follow-up periods. Incorporating advanced neurophysiological assessments, such as detailed HRV analyses or inflammatory biomarkers, could provide deeper insights into the physiological pathways influenced by fascia exercises. Additionally, qualitative methodologies, such as patient interviews, could enrich our understanding of patient experiences, adherence, and perceived benefits.

In summary, fascia exercises offer a low-cost, accessible, and integrative therapeutic option for migraine management, addressing both physical and psychological dimensions of the condition. While the results are promising, further high-quality studies are essential to establish evidence-based guidelines and integrate fascia exercises into clinical practice effectively.

## Figures and Tables

**Table 1:** Data on demographic characteristics of the participants.

Demographic Data	Treatment Group (*n* = 15) Mean ± SD	Control Group (*n* = 15) Mean ± SD	Mann–Whitney U Test	
			z Value	*p* Value
Age (years)	30.86 ± 10.32	32.80 ± 13.63	−0.354	0.723
Height (m)	1.65 ± 0.05	1.62 ± 0.06	−1.062	0.288
Weight (kg)	67.26 ± 13.97	64.46 ± 9.74	−0.727	0.467
BMI (kg/m^2^)	24.49 ± 4.21	24.71 ± 4.71	−0.145	0.885

BMI: body mass index, SD: standard deviation.

**Table 2:** Characteristics of migraine disease.

		Treatment Group (*n* = 15) *n* (%)	Control Group (*n* = 15) *n* (%)	X^2^	*p*
Migraine type	With aura	7 (46.7)	7 (46.7)	0.000	1
	Without aura	8 (53.3)	8 (53.3)		
Migraine family history	Yes	6 (40)	3 (20)	1.429	0.427
	No	9 (60)	12 (80)		

X^2^ = chi-square statistic.

**Table 3:** Comparison of the number of daily medications, number and duration of attacks, daytime and night-time pain intensity values within and between groups.

Variable	Before Treatment Mean ± SD	After Treatment Mean ± SD	Wilcoxon Signed Rank	Mean Difference (95%CI)	Effect Size	Mann–Whitney U Test ^a^
**Number of medicines per day**						0.412
Treatment group	1.20 ± 1.26	0.80 ± 0.77	0.058	0.40 (−0.00, 0.80)	0.38	
Control group	1.66 ± 1.34	1.20 ± 0.94	0.008 *	0.46 (0.18, 0.75)	0.39	
**Number of attacks**						0.202
Treatment group	1.53 ± 1.50	1.00 ± 0.84	0.033 *	0.53 (0.07, 0.99)	0.43	
Control group	2.13 ± 1.64	1.46 ± 1.30	0.004 *	0.66 (0.32, 1.00)	0.45	
**Attack duration (hours)**						0.838
Treatment group	25.46 ± 20.43	18.46 ± 13.65	**0.003** *	7.00 (1.85, 12.14)	0.40	
Control group	23.93 ± 27.74	19.73 ± 22.41	0.026 *	4.20 (−0.56, 8.96)	0.16	
**VAS Daytime**						0.983
Treatment group	7.68 ± 1.43	5.56 ± 1.36	0.001 *	2.11 (1.32, 2.90)	1.51	
Control group	7.51 ± 2.12	6.40 ± 1.59	0.007 *	1.11 (0.50, 1.72)	0.59	
**VAS Night**						0.850
Treatment group	6.18 ± 2.77	4.09 ± 1.65	0.001 *	2.09 (1.00, 3.18)	0.91	
Control group	6.80 ± 3.27	5.26 ± 2.34	0.003 *	1.54 (0.73, 2.34)	0.54	

CI: confidence interval, SD: standard deviation ^a^ comparison of mean differences in groups. * *p* < 0.05.

**Table 4:** Comparison of disability level, quality of life and sleep, and anxiety and depression levels within and between groups.

Variable	Before Treatment Mean ± SD	After Treatment Mean ± SD	Wilcoxon Signed Rank	Mean Difference (95%CI)	Effect Size	Mann–Whitney U Test ^a^
**MIDAS**						0.708
Treatment group	35.46 ± 19.75	26.53 ± 12.40	0.001 *	8.93 (4.05, 13.80)	0.54	
Control group	44.53 ± 30.19	35.46 ± 23.19	0.001 *	9.06 (4.85, 13.27)	0.33	
**24-hr-MQoLQ**						0.106
Treatment group	38.73 ± 10.57	40.66 ± 10.72	0.051	−1.93 (−4.45, 0.59)	0.18	
Control group	40.13 ± 15.90	40.73 ± 14.12	0.659	−0.60 (−2.97, 1.77)	0.03	
**PSQI**						0.624
Treatment group	8.60 ± 2.38	7.13 ± 1.95	0.003 *	1.46 (0.84, 2.08)	0.67	
Control group	8.86 ± 1.84	7.60 ± 1.88	0.002 *	1.26 (0.73, 1.79)	0.67	
**HADS-A**						0.265
Treatment group	11.20 ± 4.69	9.60 ± 3.58	0.004 *	1.60 (0.79, 2.40)	0.38	
Control group	8.86 ± 2.82	7.60 ± 2.97	0.003 *	1.06 (0.57, 1.55)	0.43	
**HADS-D**						0.068
Treatment group	7.46 ± 3.62	6.06 ± 2.89	0.006 *	1.40 (0.56, 2.23)	0.42	
Control group	6.06 ± 3.30	5.53 ± 3.22	0.084	0.53 (−0.12, 1.19)	0.16	

CI: confidence interval, SD: standard deviation ^a^ comparison of mean differences in groups. * *p* < 0.05.

**Table 5:** Comparison of the change in HRV parameters within and between groups.

Variable	Before Treatment Mean ± SD	After Treatment Mean ± SD	Wilcoxon Signed Rank	Mean Difference (95%CI)	Effect Size	Mann–Whitney U Test ^a^
**RMSSD**						0.110
Treatment group	36.71 ± 17.82	27.87 ± 11.27	0.069	8.84 (0.29, 17.40)	0.59	
Control group	36.76 ± 31.97	36.06 ± 32.97	1.00	0.70 (−3.43, 4.84)	0.02	
**SDNN**						0.254
Treatment group	55.66 ± 23.45	52.22 ± 34.71	0.069	3.43 (−6.15, 13.03)	0.11	
Control group	55.71 ± 40.76	43.35 ± 13.10	0.496	12.35 (−8.42, 33.13)	0.09	
**Mean-RR**						0.694
Treatment group	750.50 ± 109.18	767.26 ± 349.27	0.088	−17.25 (−188.04, 153.52)	0.06	
Control group	716.09 ± 140.34	674.68 ± 120.59	0.047 *	41.40 (4.93, 77.88)	0.31	
**LF/HF Ratio**						0.049
Treatment group	1.63 ± 0.76	2.18 ± 1.33	0.073	−0.55 (−1.47, 0.35)	0.50	
Control group	2.61 ± 1.71	2.70 ± 1.69	0.233	−0.09 (−0.83, 0.65)	0.05	
**LF Power**						0.468
Treatment group	472.36 ± 328.05	472.82 ± 388.72	0.650	−0.45 (−50.86, 49.95)	0.00	
Control group	347.20 ± 206.50	352.72 ± 213.42	0.394	−5.51 (−53.27, 42.24)	0.02	
**HF Power**						0.272
Treatment group	258.31 ± 133.96	264.74 ± 99.71	0.609	−6.43 (−49.53, 36.66)	0.05	
Control group	275.28 ± 184.37	222.36 ± 105.93	0.211	52.91 (−14.31, 120.14)	0.35	
**LF Peak**						0.740
Treatment group	0.20 ± 0.22	0.16 ± 0.17	0.256	0.03 (−0.07, 0.15)	0.20	
Control group	0.18 ± 0.26	0.17 ± 0.22	0.330	0.01 (−0.02, 0.04)	0.04	
**HF Peak**						0.494
Treatment group	0.26 ± 0.11	0.33 ± 0.17	0.100	−0.07 (−0.15, 0.00)	0.48	
Control group	0.31 ± 0.19	0.33 ± 0.15	0.514	−0.01 (−0.07, 0.03)	0.11	

CI: confidence interval, SD: standard deviation, ^a^ comparison of mean differences in groups. * *p* < 0.05.

**Table 6:** Comparison of patient satisfaction levels after treatment.

	Treatment Group Mean ± SD	Control Group Mean ± SD	Mann–Whitney U Test	
			Z	*p*
**Patient satisfaction**	2.53 ± 0.83	1.86 ± 0.63	−2.226	0.026 *

SD: standard deviation. * *p* < 0.05.

## Data Availability

The data that support the findings of this study are available from the corresponding author upon reasonable request.

## Abbreviations

The following abbreviations are used in this manuscript:

ANS	Autonomic nervous system
HRV	Heart rate variability
VAS	Visual analog scale
MIDAS	Migraine Disability Assessment Scale
PSQI	Pittsburgh Sleep Quality Index
HADS	Hospital Anxiety and Depression Scale
ROM	Range of motion
